# Appendicitis

**Published:** 1898-03

**Authors:** C. Alvin Strasburg

**Affiliations:** Cridersville, O.


ARTICLE XIII.

### APPENDICITIS.

BY C. ALVIN STRASBURG, M. D., CRIDERSVILLE, O.

Only a short time since, I was called to treat my second case of appendicitis. The patient was a man of more than fifty years. Found he had been vomiting. Pulse ninety-six; temperature 102°. Bowels swollen, and very tender. Urine brown. Bowels had been moving every day. Tumor in right iliac region, and a slight, stinging pain occasionally also there. Extreme rigors.

What shall I Do? was my query. Shall I operate? No! seemed to echo to me in such a way that I heeded its advice. From the rigors present it was plain that pus had already formed, and an operation would be equal to suicide for my patient. I used enemas of cool water every two hours, and upon the third administration of the enema I was pleased to see the pus follow.

The appendix had opened into the cecum, and its contents were evacuated. The pain then became less severe, and after a few hours another discharge of pus followed enema, and the rigors ceased. I advised enemas three times daily for a few days. Patient made a splendid recovery.

His internal treatment was:

℞ Ext. Aconiti fl.......................... 10 drops.
Ext. Digitalis fl.......................... 15 drops.
Glycerini.......................... 2 ounces.
Aquæ Dest.......................... 4 ounces.
M. Sig.: Teaspoonful every hour.

Upon a former occasion I used, in a more recent attack of appendicitis, equal parts of glycerine and castor oil, with very gratifying results. I should prefer this, or other simple methods, to operating, as it does not necessitate the danger of losing a surgeon's sponge, or spectacles, in the abdominal cavity. These, of course, would be hard to obtain should the patient recover.—*Medical Brief.*

